# Expression of the *KNOTTED HOMEOBOX* Genes in the Cactaceae Cambial Zone Suggests Their Involvement in Wood Development

**DOI:** 10.3389/fpls.2017.00218

**Published:** 2017-03-03

**Authors:** Jorge Reyes-Rivera, Gustavo Rodríguez-Alonso, Emilio Petrone, Alejandra Vasco, Francisco Vergara-Silva, Svetlana Shishkova, Teresa Terrazas

**Affiliations:** ^1^Departamento de Botánica, Instituto de Biología, Universidad Nacional Autónoma de MéxicoMexico City, Mexico; ^2^Departamento de Biología Molecular de Plantas, Instituto de Biotecnología, Universidad Nacional Autónoma de MéxicoCuernavaca, Mexico; ^3^Jardín Botánico, Instituto de Biología, Universidad Nacional Autónoma de MéxicoMexico City, Mexico

**Keywords:** Cactaceae, dimorphic wood, fibrous wood, KNAT7 ortholog, KNOX transcription factors, non-fibrous wood, vascular cambium, wood lignification

## Abstract

The vascular cambium is a lateral meristem that produces secondary xylem (i.e., wood) and phloem. Different Cactaceae species develop different types of secondary xylem; however, little is known about the mechanisms underlying wood formation in the Cactaceae. The *KNOTTED HOMEOBOX (KNOX)* gene family encodes transcription factors that regulate plant development. The role of class I *KNOX* genes in the regulation of the shoot apical meristem, inflorescence architecture, and secondary growth is established in a few model species, while the functions of class II *KNOX* genes are less well understood, although the *Arabidopsis thaliana* class II KNOX protein KNAT7 is known to regulate secondary cell wall biosynthesis. To explore the involvement of the *KNOX* genes in the enormous variability of wood in Cactaceae, we identified orthologous genes expressed in species with fibrous (*Pereskia lychnidiflora* and *Pilosocereus alensis*), non-fibrous (*Ariocarpus retusus*), and dimorphic (*Ferocactus pilosus*) wood. Both class I and class II *KNOX* genes were expressed in the cactus cambial zone, including one or two class I paralogs of *KNAT1*, as well as one or two class II paralogs of *KNAT3*-*KNAT4*-*KNAT5*. While the *KNOX* gene *SHOOTMERISTEMLESS* (*STM)* and its ortholog *ARK1* are expressed during secondary growth in the *Arabidopsis* and *Populus* stem, respectively, we did not find *STM* orthologs in the Cactaceae cambial zone, which suggests possible differences in the vascular cambium genetic regulatory network in these species. Importantly, while two class II *KNOX* paralogs from the *KNAT7* clade were expressed in the cambial zone of *A. retusus* and *F. pilosus*, we did not detect *KNAT7* ortholog expression in the cambial zone of *P. lychnidiflora*. Differences in the transcriptional repressor activity of secondary cell wall biosynthesis by the *KNAT7* orthologs could therefore explain the differences in wood development in the cactus species.

## Introduction

As reservoirs of pluripotent cells, meristems have played a leading role in the diversification of angiosperm growth forms. The shoot and root apical meristems maintain primary growth in plants, while the lateral meristems, comprised of the vascular cambium and cork cambium in Eudicotyledons and gymnosperms, are involved in secondary growth (Evert, 2006). The vascular cambium maintains a population of initial (stem) cells, which divide asymmetrically to generate two daughter cells; one maintains the cambial initial identity, while the other divides again and the daughters differentiate to generate secondary phloem or xylem (Groover et al., 2010; Miyashima et al., 2013). Vascular cambium derivatives are thought to have influenced speciation and diversification events (Spicer and Groover, 2010; Lucas et al., 2013). In the Cactaceae, the traits of the secondary xylem (i.e., wood) suggest that it has evolved by heterochronic processes where a change in the timing of developmental processes leads to morphological differences between species (Altesor et al., 1994). The larger species (≥1.5 m in height) in this family of succulent plants have fibrous wood with vessel elements in the xylem similar to those typically derived from vascular cambium, with a similar wood chemical composition to that of other woody dicot species (Reyes-Rivera et al., 2015). On the other hand, the wood found in smaller species is generally scarce and non-fibrous, with abundant wide-band tracheids and vessel elements similar to those typical of proto- and metaxylem. In these species, the level of wood lignification is insignificant and the lignin has a heterogeneous chemical composition (Reyes-Rivera et al., 2015). Many species of Cactaceae have dimorphic wood, where one type of wood is produced in the juvenile stages and a different structure is formed in the adult stages of development. To the best of our knowledge, this phenomenon is unique among Eudicotyledons and is related to the globose and globose-depressed growth forms of some Cactaceae species (Mauseth and Plemons, 1995). In species with dimorphic wood that produce wide-band tracheids, these always develop first, before the fibrous or parenchymatous wood is produced (Mauseth and Plemons, 1995; Loza–Cornejo and Terrazas, 2011). The mechanisms shaping the development of the vascular cambium and its derivatives in the Cactaceae are mostly unknown; nevertheless, it was suggested that the wide variation in wood anatomy of different cacti species might be attributed to a variation of gene expression patterns and gene expression level (Mauseth and Plemons, 1995; Mauseth, 2006; Landrum, 2008; Vázquez-Sánchez and Terrazas, 2011).

The interplay of diverse factors in the regulation of vascular cambium activity has been reported previously (Liu et al., 2014; Nieminen et al., 2015), and the roles of growth regulators, including auxin, cytokinin, and ethylene, in this process are well established. Later in development, genes regulating cell expansion, secondary xylem differentiation, lignification, and secondary wall deposition contribute to wood formation (reviewed in Růžička et al., 2015; Ye and Zhong, 2015; Zhong and Ye, 2015). Transcription profiling of the vascular cambium in aspen (*Populus tremula*) uncovered similarities between the gene regulatory networks operating in the shoot apical meristem and the vascular cambium. In particular, it was reported that four members of the *KNOTTED1*-*LIKE HOMEOBOX* (*KNOX)* gene family, namely *PttKNOX1*, *PttSHOOT MERISTEMLESS (PttSTM)*, *PttKNOX2*, and *PttKNOX6*, are highly expressed in both tissues (Schrader et al., 2004). Functional analysis suggests that the *Populus* orthologs of *Arabidopsis thaliana (Arabidopsis)* class I *KNOX* genes *STM* and *KNOTTED-LIKE HOMEOBOX OF ARABIDOPSIS THALIANA 1* (*KNAT1*)/*BREVIPEDICELLUS* (*BP*), *PttSTM/ARBORKNOX 1 (ARK1)/ARK1a* and *PttKNOX1/ARK2*, respectively, regulate secondary growth (Groover et al., 2006; Du et al., 2009; Liu et al., 2015). While the roles of class I *KNOX* genes in the regulation of shoot apical meristem, inflorescence architecture, and compound leaf development are well established, the functions of class II *KNOX* genes are less well understood. In general, class I transcripts are less abundant than class II *KNOX* transcripts, and are expressed in specific regions of meristems, particularly in the shoot apical meristem and leaf meristems. By contrast, class II transcripts are found in differentiating cells and all mature plant organs (Serikawa et al., 1997; reviewed by Hay and Tsiantis, 2010; Arnaud and Pautot, 2014), but not in the shoot apical meristem (Furumizu et al., 2015). Moreover, the dark-green serrated leaf phenotype of the class II *knat3 knat4 knat5* triple loss-of-function mutant in *Arabidopsis* is similar to that of the class I gain-of-function mutants (Furumizu et al., 2015), suggesting opposing functions for genes of the two classes. Here, we report that class I and class II *KNOX* genes are expressed in the cambial zone, consisting of vascular cambium and derived cells, of Cactaceae species with fibrous (*Pereskia lychnidiflora* and *Pilosocereus alensis*), non-fibrous (*Ariocarpus retusus*), and dimorphic (*Ferocactus pilosus*) wood. We also present the phylogeny of class I and class II KNOX proteins encoded in the sequenced plant genomes retrieved from the Phytozome database, confirming monophyly of class I and class II KNOX proteins, assigning the Cactaceae KNOX proteins into clades, and exploring the number of paralogs of the plant species in every KNOX clade.

## Materials and Methods

### Plant Material and Tissue Sampling

Four Cactaceae species with different wood types were used in this study: *Pereskia lychnidiflora* DC. (subfamily: Pereskoideae); *Pilosocereus alensis* (F. A. C. Weber) Byles & G. D. Rowley (subfamily: Cactoideae, tribe: Cereeae); *Ariocarpus retusus* Scheidw (Cactoideae, tribe: Cacteae); and *Ferocactus pilosus* (Galeotti ex Salm-Dyck) Werderm. (Cactoideae, Cacteae). Samples were collected from the cambial zone of adult individuals growing in Mexico. For the tall species (*F. pilosus*, *P. lychnidiflora*), the cambial zone was harvested from one individual of both species in the field (*F. pilosus* at the Mexican Plateau, San Luis Potosí state, location 23°19′31′′N; 100°33′30′′W, voucher TT890 MEXU; and *P. lychnidiflora* at the Isthmus of Tehuantepec, Oaxaca state, location 16°22′52′′N; 95°19′03′′W, voucher TT966 MEXU), with samples collected during the rainy season to ensure that the vascular cambium was active. All surrounding tissues including the cortex were removed as described by Liu et al. (2014). The cambial zone was peeled off with a disposable microtome knife and immediately frozen in liquid nitrogen. One whole individual of both *A. retusus* and *P. alensis* were placed in pots containing their native soil and transported from their natural habitat to the laboratory (*A. retusus* from the Mexican Plateau, Nuevo León state, location 23°23′39′′N; 100°21′57′′W, voucher SA1976 MEXU; and *P. alensis* from the Pacific coast, Michoacán state, location 18°46′41′′N; 103°08′05′′W, voucher JRR3 MEXU). Species identification was confirmed by S. Arias [Instituto de Biología, Universidad Nacional Autónoma de México (IB, UNAM)], the leading taxonomy specialist of the Cactaceae family in Mexico. The cambial zone was harvested as described above. The cambial zone samples were stored at −80°C until RNA extraction. For expression analysis by RT-PCR, one adult plant of *A. retusus, F. pilosus*, and *P. lychnidiflora*, one 2-year-old juvenile plant of the first two species, and two 5-year-old young individuals of *A. retusus*, previously collected from the same locations, were donated by the Botanical Garden IB, UNAM. For the root tip transcriptome of *Pachycereus pringlei* (S. Watson) Britton & Rose, plants were germinated from seeds collected near Bahia Kino, Sonora state, and donated by F. Molina-Freaner and J. F. Martínez-Rodríguez (Instituto de Ecología, campus Hermosillo, UNAM).

For the wood maceration, fine wood chips (2 mm thickness) were obtained every 5 mm, from the region closest to the pith (young wood) to the section closest to the vascular cambium (mature wood), and each region was processed separately. The non-fibrous *A. retusus* samples were placed in 2-mL microcentrifuge tubes filled with Franklin solution (5:1:4 acetic acid:hydrogen peroxide:water; Ruzin, 1999), while the dimorphous *F. pilosus* samples and the fibrous *P*. *alensis* and *P*. *lychnidiflora* were placed into tubes containing Jeffrey solution (equal volumes of 10% chromic acid and 10% nitric acid; Berlyn and Miksche, 1976). For each species, 0.2 g of the tissue was used. The samples were then incubated at 56°C for 4 h (non-fibrous wood) and 24 h (dimorphic and fibrous wood). Additionally, all samples were sonicated in a Branson 200 ultrasonic cleaner (Branson Ultrasonics, Danbury, CT, USA) until completely macerated, washed with water, and dehydrated using a series of ethanol washes at 50, 70, 90%, and absolute concentrations. The *A. retusus* macerations were stained with a 0.1% aqueous solution of toluidine blue for 12 h (Ruzin, 1999) and mounted onto slides. The macerations of the wood samples from another three species were stained with Safranin for 2 h and mounted onto slides using synthetic resin (Entellan, Merck Millipore, Darmstadt, Germany). All wood elements were photographed using a BX51 optical microscope (Olympus Corporation, Tokyo, Japan) and the images were analyzed using Image-Pro Plus v. 6.1 software (Media Cybernetics, Inc., Bethesda, MD, USA).

### RNA Extraction, cDNA Synthesis, and PCR Amplification

Total RNA was extracted using TRIzol Reagent (Invitrogen, Carlsbad, CA, USA), according to the manufacturer’s protocol, including the optional step of centrifugation before the separation of phases, or using the Spectrum Plant Total RNA Isolation Kit (Sigma–Aldrich, St. Louis, MO, USA). The cDNA was synthesized using SuperScript II Reverse Transcriptase (Invitrogen), according to the manufacturer’s instructions. Degenerate PCR primers were used to amplify the *KNOX* genes. Primers for amplifying putative cacti orthologs of *KNAT3* were designed (KNAT3_Cact_F: 5′-GAGAGRAATAATGGCWTATCATC-3′ and KNAT3_Cact-R: 5′-CCTTCTGGTTCTACTTCCCTC-3′) based on the alignments of nucleotide sequences encoding the class II KNOX proteins *KNAT3* (*Arabidopsis*) and its closest homologs from *Beta vulgaris* and *Pachycereus pringlei.* For the Cactaceae orthologs of *KNAT1*, primers designed by Du et al. (2009) were used. PCR products were purified with Sephadex Centri-sep columns (Thermo Fisher Scientific, Waltham, MA, USA) as instructed by the manufacturer. The amplified and purified products were sequenced in a 3500xL Genetic Analyzer sequencer (Applied Biosystems, Foster City, CA, USA) using the PCR primers. Platinum Taq polymerase (Thermo Fischer Scientific, Waltham, MA, USA) was used for PCR reactions. Primers used for RT-PCR are listed in **Supplementary Table S1**. RNA-seq was performed at the Beijing Genome Institute, Hong Kong; the vascular cambium and root tip transcriptomes were *de novo* assembled using Trinity v. 2.2^1^ and CLC Genomic Workbench v. 7.5 (Qiagen^2^), respectively.

### Sequence Alignment and Phylogenetic Analysis

KNOX-like protein sequences were retrieved from the Phytozome database^3^ (v. 11; last accessed on May 9, 2016) using the PhytoMine tools. All proteins with KNOX (IPR005540 [InterProscan definition]/PF03790 [Pfam definition] and IPR005541/PF03791), ELK (IPR005539/PF03789), and HD domains (IPR009057/PF05920) were retrieved. *B. vulgaris* sequences were retrieved using tBLASTn from the RefBeet v. 1.2 genome assembly (The *Beta vulgaris* resource^4^, Dohm et al., 2013) using a BLOSUM80 substitution matrix (Henikoff and Henikoff, 1992; at the time, *B. vulgaris* was the only species from the order Caryophyllales with a sequenced genome). Chimeric sequences and those from the early release genomes were discarded. After that, KNOX protein sequences from the tree species *Betula luminifera* and *Juglans nigra* were added. For Cactaceae species, the KNOX protein sequences used were deduced from the amplified and sequenced PCR fragments (see previous section), from our RNA-seq data and the *de novo* assembly of the cambial zone transcriptome of the four species reported in this work (the same RNA samples were used as starting material for the cambial zone transcriptome assembly), from the root tip transcriptome of the Cactoideae subfamily species *Pachycereus pringlei* (the analysis of the transcriptomes will be reported elsewhere), and from the recently published *Lophophora williamsii* transcriptome (Ibarra-Laclette et al., 2015). The resulting sequences were aligned with Clustal Omega^5^ and the alignment file was manually edited. Alignment positions with more than 30% gaps were not included in the analysis. The identity and similarity values (%) were obtained from a pairwise alignment (Needle-EMBOSS^6^), with a complete gap deletion for each pair.

The phylogeny was reconstructed with MEGA7 (Kumar et al., 2016). A maximum likelihood algorithm based on the JTT substitution model (Felsenstein, 1981) was used to resolve the phylogenetic relationship of the KNOX proteins derived from the Cactaceae species and the plant species with sequenced genomes. The resulting topology was statistically tested with a 1000 replicate bootstrap for both the complete and the selected datasets. The NWK files were visualized and edited on FigTree^7^ (v. 1.4.2). The BELL 1 protein of *Arabidopsis* (AT5G41410.1) was used as the outgroup. Nucleotide sequences reported in this work were deposited in GenBank under the accession numbers KX891335-KX891338 for *F. pilosus*; KX891339-KX891343 and KX891349 for *A. retusus*; KX891344-KX891346 for *P*. *lychnidiflora*; KX891347 and KX891348 for *P. alensis*; and KX870027-KX870031 for *P. pringlei*.

## Results

### Morphological Analysis of Xylem Cells

**FIGURE 1:**
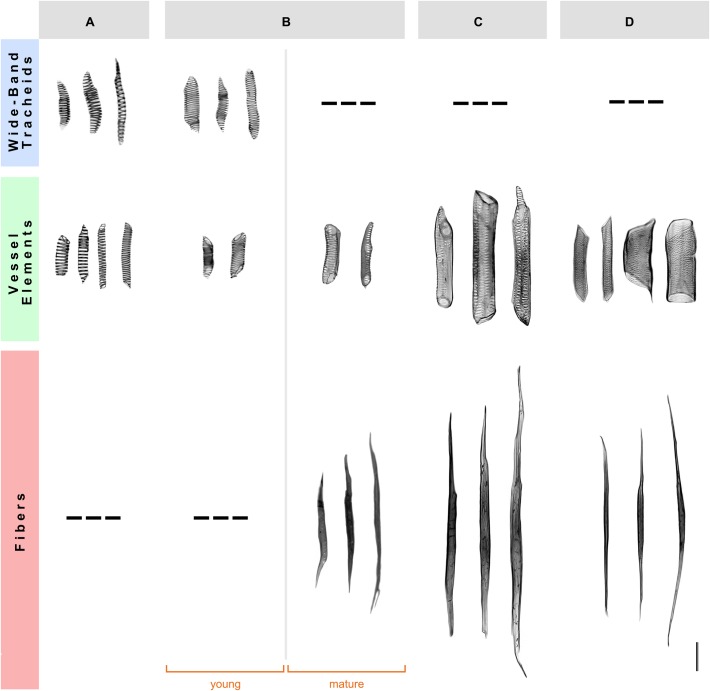
**Morphology of the secondary xylem cells in four species of Cactaceae. (A)** Non-fibrous wood of *Ariocarpus retusus*, from a 15-cm tall specimen. **(B)** Dimorphic wood of *Ferocactus pilosus*, from a 90-cm tall specimen. **(C)** Fibrous wood of *Pilosocereus alensis*, from a 1.5-m tall specimen. **(D)** Fibrous wood of *Pereskia lychnidiflora*, from a 4-m tall specimen. Only mature wood is illustrated in **(A,C,D)**, as no differences were observed between young and mature wood of these species. Scale bar: 100 μm.

To examine the detailed cellular features of the secondary xylem in the four species studied, we performed a morphological analysis of xylem cells using wood macerations. The young xylem of *A. retusus* with non-fibrous wood and *F. pilosus* with dimorphic wood forms wide-band tracheids and vessel elements with annular and helical secondary wall patterns formed during early development. The mature fibers and vessel elements with pseudoscalariform and alternate pitting were formed in adult plants during late development. No differences were observed between young and mature wood of *P. alensis* and *P. lychnidiflora*, with both having fibrous wood. The tracheary elements of *A. retusus* and young *F. pilosus* had a low proportion of lignified cell wall, similar to those of proto- and metaxylem (i.e., primary xylem), whereas vessel elements and fibers of mature *F. pilosus*, *P. alensis*, and *P. lychnidiflora*, had a higher proportion of lignified cell walls. This analysis allowed the unequivocal identification of each secondary xylem cell type and its cell wall features (**Figure 1**) and thus confirmed the wood types we previously identified using an anatomical analysis of wood sections (Reyes-Rivera et al., 2015). The variability in size and pitting of vessel elements associated with the presence of fibers was found in species with dimorphic and fibrous wood.

### *KNOX* Family Genes Are Expressed in the Cactaceae Cambial Zone

**FIGURE 2:**
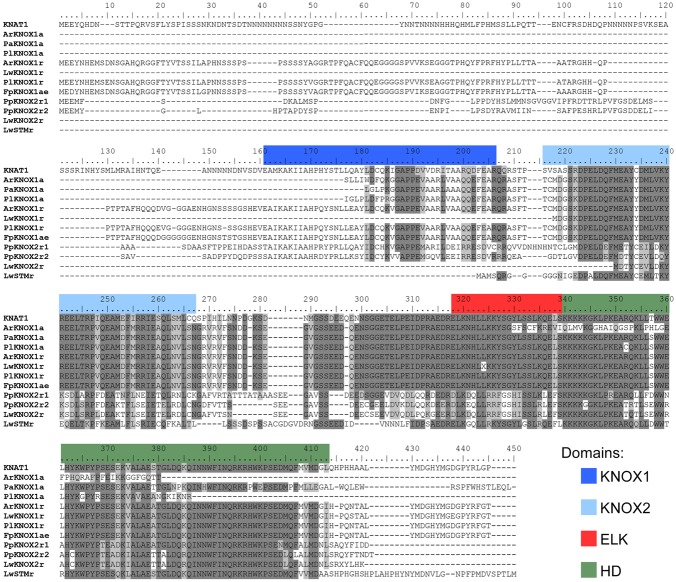
**Alignment of the deduced amino acid sequences of the class I KNOX proteins of Cactaceae.** The amino acids are highlighted with a grayscale background according to their identity and similarity values only if the coverage in the alignment position is higher than 55%. As part of the KNOX1 domain is not covered in the incomplete protein sequences, the amino acids in the other proteins are not highlighted, despite a high percentage of identity/similarity. Domains were defined with InterProscan. The *Arabidopsis* KNAT1 sequence was included as a reference.

To explore the involvement of the *KNOX* family in the enormous variability of wood morphologies in the Cactaceae, we looked for orthologs of these genes expressed in the Cactaceae cambial zone, which comprises the vascular cambium and recently derived cells. RT-PCR using degenerate primers for the class I *KNOX* genes resulted in a major amplification product of the predicted molecular weight in the four Cactaceae species studied. The amino acid sequences inferred from the amplified and sequenced fragments of these genes (*ArKNOX1a* from *Ariocarpus retusus*, *FpKNOX1a* from *Ferocactus pilosus*, *PaKNOX1a* from *Pilosocereus alensis*, and *PlKNOX1a* from *Pereskia lychnidiflora*) correspond to nearly 70% of the ARK2 and KNAT1 protein sequences of *Populus* and *Arabidopsis*, respectively, including part of the KNOX1 domain and the entirety of the KNOX2, ELK, and HD domains [**Figure 2** (The last letters in the gene names refer to: “a,” amplification as a method of gene isolation; “e,” the inferred coding sequence extended after alignment with sequences resulting from the RNA-seq; and “r,” RNA-seq as a method of gene identification)].

**FIGURE 3:**
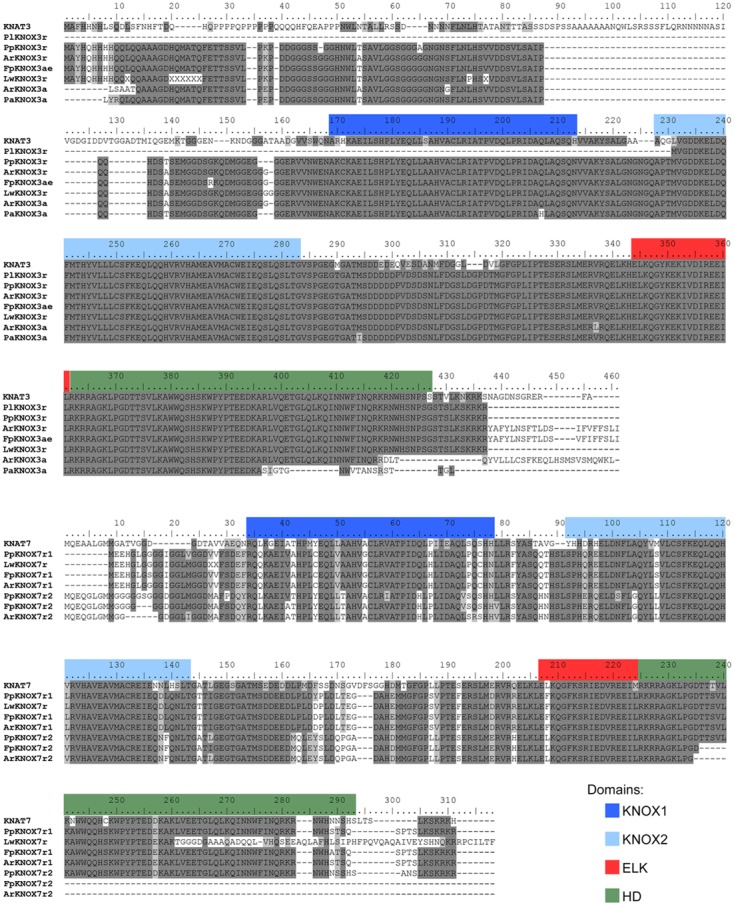
**Alignment of the deduced amino acid sequences of the class II KNOX proteins of Cactaceae and some class II KNOX proteins of *Arabidopsis*.** The amino acid scores are highlighted with a grayscale background according to their identity and similarity only if the coverage in the alignment position is higher than 55%. As part of the KNOX1 domain is not covered in the incomplete protein sequences, the amino acids in the other proteins are not highlighted, despite a high percentage of identity/similarity. Domains were defined with InterProscan.

Fragments of class II *KNOX* genes were successfully amplified by RT-PCR with degenerate primers from the three species from the Cactoideae subfamily, but were not isolated from *P. lychnidiflora*. The amino acid sequences inferred from the amplified and sequenced fragments of these three genes, *ArKNOX3a*, *FpKNOX3a*, and *PaKNOX3a*, cover nearly 95% of the KNAT3 protein sequence, including the KNOX1, KNOX2, and ELK domains, as well as most of the HD domain (**Figure 3**).

**FIGURE 4:**
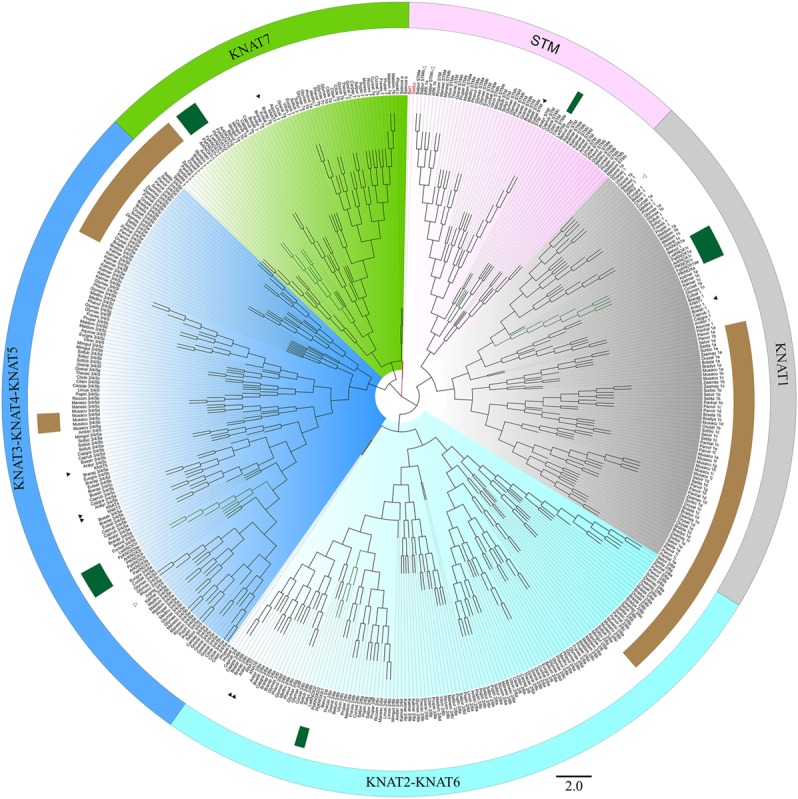
**Maximum likelihood phylogenetic tree of KNOX proteins.** Phylogeny of the complete dataset, incorporating the deduced proteins of the five Cactaceae species analyzed in this study; the deduced proteins from the 44 sequenced plant genomes retrieved from the Phytozome database and the *Beta vulgaris* genome; sequences from *Betula luminifera* and *Juglans nigra* trees; and proteins deduced from the *Lophophora williamsii* transcriptome (Ibarra-Laclette et al., 2015). The gene identifiers for each sequence are listed in **Supplementary Table S2**. The branches for the Cactaceae and *Beta vulgaris* sequences are represented in green and indicated with green ribbons. The sequences from monocots (Poaceae and Musaceae) are indicated by brown ribbons. The BEL1 protein from *Arabidopsis thaliana* was used to root the tree and is indicated in red. The KNOX protein from the unicellular green alga *Ostreococcus lucimarinus* at the base of the tree is also indicated in red. The five main clades are named according to their putative *Arabidopsis* ortholog, indicated by a black arrowhead. The class I KNOX proteins belong to the KNAT1 (gray), KNAT2-KNAT6 (cyan), and STM (pink) clades. The class II KNOX proteins belong to the KNAT3-KNAT4-KNAT5 (blue) and KNAT7 (green) clades. *Populus* class I *KNOX* genes and the *Juglans* class II gene mentioned in the discussion are the only *KNOX* genes reported to be involved in wood formation in these tree species and are indicated by open arrowheads. The scale bar depicts the number of substitutions per sequence.

**FIGURE 5:**
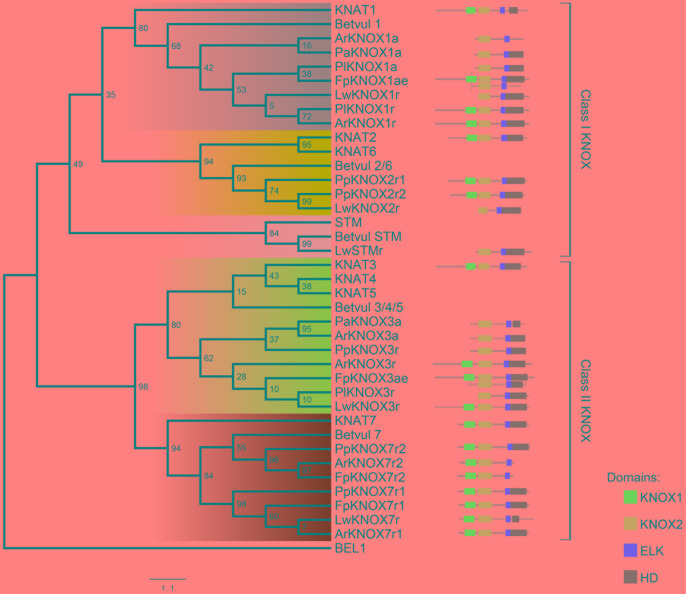
**Phylogenetic tree of the selected dataset of KNOX proteins from *Arabidopsis*, the Cactaceae, and *Beta vulgaris*.** The class I KNOX proteins belong to the KNAT1 (gray), KNAT2-KNAT6 (cyan), and STM (pink) clades. The class II KNOX proteins belong to the KNAT3-KNAT4-KNAT5 (blue) and KNAT7 (green) clades. The domain positions for Cactaceae sequences are displayed on the right alongside the KNAT ortholog. The ELK domain in KNAT7 proteins is recognized by InterProscan but not by PFAM tools; therefore, the InterProscan database was used for the domain definition. For *FpKNOX1ae* and *FpKNOX3ae*, the portion of the original amplified sequence is shown below the assembled sequence. Note that it was not possible to obtain the full KNOX1 domain sequence for the Cactaceae sequences ending in “a”; therefore, their phylogenetic position might be slightly changed when the complete protein sequence is available. Bootstrap values from 1000 trees are displayed at the nodes. The scale depicts the number of substitutions per sequence.

In addition to the seven transcripts mentioned above, eight more *KNOX* transcripts were *de novo* assembled from our RNA-seq data of the cambial zone samples of three Cactaceae species, *A. retusus*, *F. pilosus*, and *P. lychnidiflora*. The amino acid sequences deduced from the amplified transcripts of *F. pilosus* were later extended by alignment with the assembled transcripts, and were therefore renamed *FpKNOX1ae* and *FpKNOX3ae* (**Figures 2–5**). Expression of all *KNOX* transcripts in the cambial zone of *A. retusus*, *F. pilosus*, and *P. lychnidiflora* was confirmed by RT-PCR (**Supplementary Figure S1**). Remarkably, in the cambial zone of 2-year-old juvenile plants of both *A. retusus* and *F. pilosus*, one of the two orthologs of *KNOX3*, namely, *ArKNOX3a* and *FpKNOX3ae*, and of *KNOX7*, namely, *ArKNOX7r1* and *FpKNOX7r2*, were expressed. No expression of *KNOX1* orthologs was detected in young plants. At this stage, only very scarce accumulation of wood, represented by wide-band tracheids and vessel elements, was detected in these species. From the six *KNOX* genes expressed in the vascular cambium of adult *A. retusus* plant, expression of only *ArKNOX7r1* was detected in the tubercle of 5-year-old plant (data not shown).

### Phylogenetic Analysis of the *KNOX* Family

Initially, 524 KNOX-like protein sequences were retrieved from the Phytozome database. Chimeric sequences and those from draft genome releases were filtered out, and the inferred protein sequences of Cactaceae, the trees *B. luminifera* and *J. nigra*, as well as those retrieved from the *B. vulgaris* genome, were included. The resulting matrix contained 478 aligned protein sequences belonging to 45 species with sequenced genomes, six Cactaceae species, and two species of tree. The identifiers of the sequences retrieved from the Phytozome database are listed in **Supplementary Table S2**. The maximum likelihood phylogeny grouped the KNOX proteins into five main clades (**Figures 4**, **5**), which were named according to their putative *Arabidopsis* ortholog: i.e., the KNAT1 clade (gray), KNAT2-KNAT6 clade (cyan), STM clade (pink), KNAT3-KNAT4-KNAT5 clade (blue), and KNAT7 clade (green). The first three clades belong to the class I KNOX and the last two belong to the class II KNOX proteins. **Figure 5** depicts the phylogenetic relationships of the subset of KNOX proteins from *Arabidopsis*, the Cactaceae, and *B. vulgaris. Arabidopsis* was used as the reference species, with *B. vulgaris* from order Caryophyllales, to which the Cactaceae family belongs, included as the closest sister taxon with a sequenced genome.

The molecular phylogenetic analysis confirmed that the four Cactaceae class 1 *KNOX* genes amplified from the cambial zone by RT-PCR with degenerate primers (*ArKNOX1a*, *FpKNOX1ae*, *PaKNOX1a*, and *PlKNOX1a*) are part of the KNAT1 clade (**Figures 4**, **5**); moreover, two of the *de novo* assembled transcripts, *ArKNOX1r* and *PlKNOX1r*, also belonged to the KNAT1 clade. The class II *ArKNOX3a*, *FpKNOX3ae*, and *PaKNOX3a* genes expressed in the vascular zone of the three species fall into the KNAT3-KNAT4-KNAT5 clade, along with two other class II *KNOX* genes, *ArKNOX3r* and *PlKNOX3r* (the only *Pereskia* class II gene identified in this study). Four *de novo* assembled transcripts, *ArKNOX7r1*, *ArKNOX7r2*, *FpKNOX7r1*, and *FpKNOX7r2*, belong to the KNAT7 clade (**Figures 4**, **5** and **Table 1**). The identity and similarity percentages for the proteins encoded by the identified Cactaceae genes are shown in **Supplementary Figure S2**.

**Table 1:** Number of *KNOX* genes from this study expressed in the cambial zone, root tip, and shoot/root of Cactaceae species.

		Number of *KNOX* orthologs				
		Class I clades			Class II clades	
Species	Genes expressed in	*STM*	*KNAT1*	*KNAT2-KNAT6*	*KNAT3-KNAT4-KNAT5*	*KNAT7*
*Pereskia lychnidiflora*	Cambial zone	0	2	0	1	0
*Ariocarpus retusus*	Cambial zone	0	2	0	2	2
*Ferocactus pilosus*	Cambial zone	0	1	0	1	2
*Pilosocereus alensis*^a^	Cambial zone	0	1	0	1	0
*Pachycereus pringlei*	Root tip	0	0	2	1	2
*Lophophora williamsii*^b^	Shoot and root	1	1	1	1	1

*^a^ No RNA-seq data available. ^b^ For comparison, the data from Ibarra-Laclette et al. (2015) were used.*

Of the five *KNOX* transcripts detected in the *P. pringlei* root tip, both of the class I *KNOX* genes were found in the KNAT2-KNAT6 clade, one class II sequence was attributed to the KNAT3-KNAT4-KNAT5 clade, and two more belonged to the KNAT7 clade. Among the five *KNOX* genes identified in the recently published *L. williamsii* transcriptome (Ibarra-Laclette et al., 2015), one was found in each of the five clades (**Figures 4**, **5** and **Table 1**). In the global KNOX phylogeny (**Figure 4**), *B. vulgaris* sequences were always resolved as sister to the Cactaceae sequences. In the phylogeny for the subset of KNOX proteins (**Figure 5**), *B. vulgaris* class I KNOX proteins were resolved as sister to the Cactaceae sequences in all three clades, while this was not the case for two clades of class II KNOX. The only *B. vulgaris* paralog of KNAT3, KNAT4, and KNAT5 fell in the subclade of the *A. thaliana* sequences. Within the KNAT7 clade, the *B. vulgaris* sequence represented the sister sequence for the Cactaceae KNOX7r2 subclade, whereas the KNOX7r1 sequences grouped as a separate subclade. The possible reasons of this small inconsistency could be the incomplete sequences of many Cactaceae proteins, as well as the very restricted number of sequences in this phylogeny (**Figure 5**).

## Discussion

Although, it is well established that some KNOX transcription factors are important for the maintenance of the shoot apical meristem, their role in vascular cambium activity and secondary growth is less well understood. Four class I *KNOX* genes were reported to be expressed in the vascular cambium of *P. tremula* (Schrader et al., 2004; Du et al., 2009), while the expression of a class II *KNOX* gene was detected in the vascular cambium of *J. nigra* (Huang et al., 2009). It was therefore of particular interest to determine whether genes of both classes are expressed in the vascular zones of cacti, and whether different paralogs are expressed in species producing different types of wood. By performing RT-PCR with degenerate primers in the four Cactaceae species (*A. retusus*, *F. pilosus*, *P. lychnidiflora*, and *P. alensis*) and RNA-seq with subsequent *de novo* transcriptome assembly for three of these species, we have found, and consequently confirmed by RT-PCR (**Supplementary Figure S1**), that the transcripts of both class I and class II *KNOX* genes are present in the cambial zone of the adult individuals in all four species (**Table 1**). The possibility exists that other *KNOX* genes could be expressed at very low levels in the Cactaceae vascular zone, impeding successful *de novo* assembly. Genome sequencing of species from the Cactaceae family will facilitate a more detailed analysis of the *KNOX* expression patterns. To annotate the Cactaceae KNOX sequences, a phylogenetic tree was constructed that included KNOX sequences from sequenced angiosperm genomes from the Phytozome database and from the *B. vulgaris* genome as a species from the order Caryophyllales, to which the Cactaceae family belongs. Expression of all *KNOX* genes in the cambial zone of adult *A. retusus*, *F. pilosus*, and *P. lychnidiflora* plants was confirmed by RT-PCR (**Supplementary Figure S1**). Meanwhile, in the tubercle of 5-year-old *A. retusus* plant, we detected expression of only one of the six *ArKNOX* genes reported in this work, *ArKNOX7r1*. Expression of two *KNOX* genes, the orthologous *ArKNOX7r1/FpKNOX7r1* and *ArKNOX3a/FpKNOX3ae*, was detected in the vascular cambium of juvenile *A. retusus* and *F. pilosus* plants, when they were just starting to accumulate wood. This finding further suggests that the *KNOX* genes, reported in this work, are involved in the formation of mature wood, and that the *KNOX7r1* paralog has broader functions in Cactaceae development.

The six class I *KNOX* genes found to be expressed in the cambial zone of the four cactus species are putative orthologs of the *Arabidopsis BP/KNAT1* gene, as they belong to the KNAT1 clade. The *Populus* gene *ARK2/PttKNOX1*, which is expressed in the vascular cambium and developing xylem (Schrader et al., 2004; Du et al., 2009), also belongs to this clade (white arrowhead in **Figure 4**). Previous work has suggested that *ARK2* is involved in vascular cambium activity and secondary growth; *Populus* plants constitutively overexpressing *ARK2* had a wider cambial zone and decreased differentiation of the lignified secondary xylem, while the downregulation of *ARK2* resulted in the early differentiation of lignified secondary xylem cells (Du et al., 2009).

Importantly, we did not find putative orthologs of the *Arabidopsis STM* and *KNAT2-KNAT6* genes expressed in the Cactaceae cambial zone. By contrast, both the *ARK1a* and *ARK1b* paralogs in *Populus* (orthologous to *STM*) are shown to be expressed in the vascular cambium (Popgenie database^8^). *ARK1* overexpression in a hybrid *Populus* resulted in pleiotropic phenotypes, including the slower differentiation of cambium-derived cells (Groover et al., 2006). While the *KNAT1* ortholog *ARK2* is expressed in both the vascular cambium and the developing xylem of *Populus*, *ARK1a* was shown to be downregulated in non-meristematic secondary vascular tissues (Groover et al., 2006), thus showing more similarity to the *STM* expression pattern in the shoot apex. *STM* expression was previously also detected in the *Arabidopsis* inflorescence stems during induced secondary growth (Ko and Han, 2004). As expected, we did not find a *STM* ortholog in the *P. pringlei* root tip transcriptome, while two *P. pringlei* paralogs orthologous to KNAT2-KNAT6 were expressed in the root tip (**Figures 4**, **5** and **Table 1**). The *STM* putative ortholog was present in the *L. williamsii* shoot and root transcriptome, suggesting that *STM* might be expressed in some Cactaceae tissues, most probably in the shoot apical meristem. The absence of putative *STM* orthologs in the cambial zone transcriptome suggests possible differences in the vascular cambium genetic regulatory network in the Cactaceae versus *Arabidopsis* and *Populus*. Interestingly, monocots do not have orthologs of *Arabidopsis STM*; the grass genes involved in shoot apical meristem maintenance (maize: *KNOTTED1* and *ROUGH SHEATH1*; rice: *ORYZA SATIVA HOMEOBOX1* (*OSH1)* and *OSH15*; Tsuda et al., 2011; Bolduc et al., 2014) belong to the KNAT1 clade (**Figure 4**). Moreover, monocot *KNOX* genes clustered as a single subclade within the KNAT1, KNAT2-KNAT6, and KNAT7 clades, while within the KNAT3-KNAT4-KNAT5 clade two subclades, one from Poaceae and another from Musaceae, were present (brown ribbons on **Figure 4**).

**FIGURE 6:**
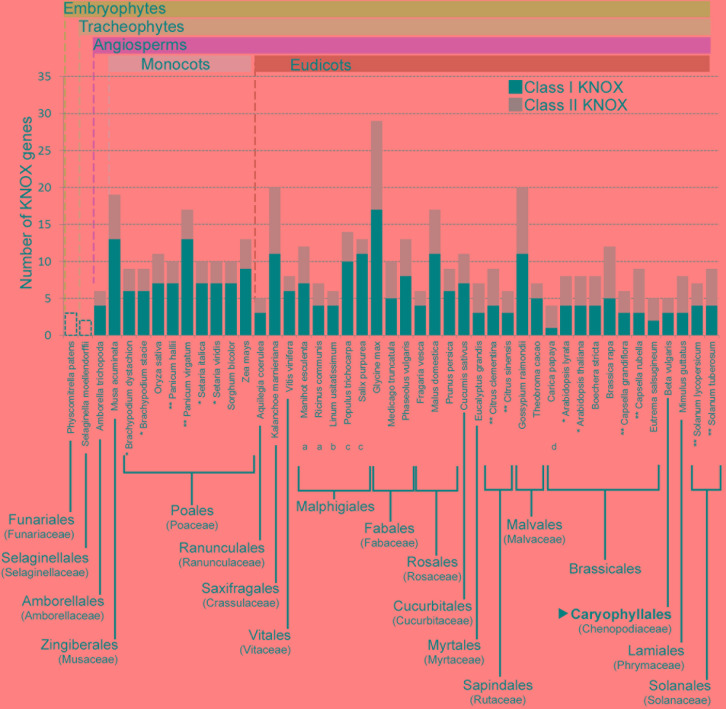
**Number of *KNOX* genes in species from different plant taxa.** The number of both class I and class II *KNOX* genes are shown for the angiosperms, while for *Physcomitrella patens* and *Selaginella moellendorffii*, only the total numbers of *KNOX* genes are indicated. When all the species from an order belonged to the same family, the family is indicated in parentheses below the order. Where different families of an order are depicted, they are indicated with lowercase letters below the species name. For the order Malpighiales, the included families are: (a) Euphorbiaceae, (b) Linaceae, and (c) Salicaceae. For the order Brassicales, one species belongs to the (d) Caricaceae family, while the rest belong to the Brassicaceae family. The order Caryophyllales, to which the Cactaceae family belongs, is depicted in bold and indicated by a black triangle. Cactaceae species are not included, because there are no Cactaceae species with sequenced genomes.^∗^ Species from the same genus with the same number of *KNOX* genes. ^∗∗^ Species from the same genus with different numbers of *KNOX* genes. Note that the number of *KNOX* genes belonging to class I, class II, or both, can be different.

For the three species used for RNA-seq and the *de novo* transcriptome assembly, two class I paralogs were identified in the vascular zone of *A. retusus* and *P. lychnidiflora*, while only one was identified in *F. pilosus* (**Figures 2**, **4**, **5** and **Table 1**). The missing class I *KNOX* paralog could have been lost from *F. pilosus* as an evolutionary developmental process, enabling speciation after a gene duplication in the Cactaceae, a phenomenon that has been well documented in other families. Using data from sequenced genomes (**Supplementary Table S2**), we showed that the number of class I and class II *KNOX* genes in a moss, a lycophyte, and 43 angiosperm species varies significantly, particularly between Eudicotyledonous species (**Figure 6**). As there is still no Cactaceae species with a sequenced genome, Cactaceae were not included in this analysis. All of the analyzed angiosperm species have several class II genes, and all but *Carica papaya* have more than one class I gene. There are 29 *KNOX* genes in *Glycine max* (17 class I and 12 class II genes) but only four in *Carica* (one class I and three class II genes). Furthermore, different species can have a different number of paralogs in every clade (**Supplementary Table S2** and **Figure 5**). The number of *KNOX* genes can also vary within species of the same family; for instance, within diploid species of the Brassicaceae family (which includes the largest number of species with sequenced genomes), the number of *KNOX* genes varies from five in *Eutrema salsugineum* to 12 in *Brassica rapa*. Even species within a single genus can have variable numbers of *KNOX* genes; while the *Arabidopsis* species *A. thaliana* and *A. lyrata* both possess eight *KNOX* genes, there are six and nine genes in the *Capsella grandiflora* and *C. rubella* genomes, respectively (**Figure 6** and **Supplementary Table S2**).

We found that two putative orthologs of the *Arabidopsis* class II *KNOX* paralogs *KNAT3*, *KNAT4*, and *KNAT5* were expressed in the cambial zone of *A. retusus*, but just one was expressed in the cambial zone of *F. pilosus* and *P. lychnidiflora* (**Figures 3–5** and **Table 1**). Only one putative paralog expressed in the root tip of *P. pringlei* belonged to this class. One paralog from the published shoot and root transcriptome of *L. williamsii* (Ibarra-Laclette et al., 2015) also belongs to this class. Remarkably, among the three species used for cambial zone transcriptome assembly, two paralogs in *A. retusus* and *F. pilosus* belonged to the KNAT7 clade, whereas no putative *KNAT7* ortholog was found in the cambium of *P. lychnidiflora* (*P. alensis* was not considered as the transcriptome assembly was not performed for this species). KNAT7 is a transcriptional repressor of secondary cell wall biosynthesis (Liu et al., 2014). *P. lychnidiflora* has fibrous wood with significant cell wall lignification, *A. retusus* has non-fibrous wood with abundant wide-band tracheids and less lignification, while in the dimorphic wood of *F. pilosus*, the wide-band tracheids develop before more lignified cells. Our findings therefore suggest that the repressor activity of the *KNAT7* orthologs during secondary cell wall biosynthesis could explain the differences in wood type among these species, including the lower lignification level observed in *A. retusus* and *F. pilosus*.

We found that the transcripts of both *KNOX* gene classes are expressed in the cambial zone of the four Cactaceae species studied, and therefore could be expressed either in different cell types or in the same cell type within the vascular zone. Several KNOX proteins have been shown to selectively move from one cell layer to another (reviewed by Han et al., 2014), suggesting that even if the class I and II genes are transcribed in different cell types, the encoded proteins could coexist in the same cell as a result of protein trafficking. In any case, as no evidence of mutual repression was found when class I and class II *KNOX* loss-of-function mutations of *Arabidopsis* were combined (Furumizu et al., 2015), KNOX proteins belonging to different classes could exert their functions within the same cell. KNOX proteins are known to interact with a sister group of proteins from the BEL1-LIKE-HOMEODOMAIN (BHL, or BELL from the founding *BELL 1* gene) family; both KNOX and BELL proteins belong to the TALE (three amino acid loop extension) superclass of homeobox proteins. The KNOX-BELL heterodimer formation affects its cellular localization and the KNOX target selection (reviewed by Di Giacomo et al., 2013; Arnaud and Pautot, 2014). From heterologous expression experiments it was proposed that particular KNOX proteins could interact with different BELL partners leading to numerous combinations with distinct activities, and thus regulating different sets of targets, including transcription factors and hormonal pathways, and ultimately influence multiple plant developmental processes. Recently, however, a more specific selectivity was suggested for the *in vivo* KNOX-BELL interactions (Furumizu et al., 2015), which could also enhance the properties of each heterodimerization partner; thus, the interaction between the transcriptional repressor KNAT7 and its partner BHL6 could enhance their activity in repressing genes involved in secondary cell wall biosynthesis (Liu et al., 2014).

Knowledge of the processes of duplication and subfunctionalization of the regulatory genes helps us understand the evolution of the different aspects of plant development (Pires and Dolan, 2012), including the evolution of vascular development. Our work contributes to the elucidation of the mechanisms by which different wood types are formed in the Cactaceae, and provides insight into the evolutionary history of *KNOX* genes in different angiosperm species and their role in the speciation of land plants.

## Author Contributions

TT, JR-R, AV, FV-S, GR-A, and SS designed the work; TT, JR-R, EP, AV, GR-A, and SS performed the experiments and analyzed the data; GR-A and JR-R prepared the figures; and TT and SS wrote the manuscript. All the authors have read and approved the manuscript.

## Conflict of Interest Statement

The authors declare that the research was conducted in the absence of any commercial or financial relationships that could be construed as a potential conflict of interest.

## Footnotes

1 https://github.com/trinityrnaseq/trinityrnaseq/releases

2 https://www.qiagenbioinformatics.com/products/clc-genomics-workbench/

3 http://phytozome.jgi.doe.gov

4 http://bvseq.molgen.mpg.de/index.shtml

5 http://www.ebi.ac.uk/Tools/msa/clustalo/

6 http://www.ebi.ac.uk/Tools/psa/emboss_needle/

7 http://tree.bio.ed.ac.uk/

8 http://popgenie.org/

## Supplementary Material

FIGURE S1 **Class I and II KNOX transcript expression detected in cambial zone samples by end point RT-PCR**.

FIGURE S2 **The identity and similarity matrix of the deduced KNOX proteins identified in this study. (A)** Class I proteins. **(B)** Concatenated KNOX1, KNOX2, ELK, and HD domains of class I proteins. **(C)** Class II proteins. **(D)** Concatenated KNOX1, KNOX2, ELK, and HD domains of class II proteins.

TABLE S1 **PCR primers used in this study**.

TABLE S2 **The identifiers of the KNOX sequences used for molecular phylogenetic analysis**.
