# Supplementary figures and images for: Expression of the KNOTTED HOMEOBOX Genes in the Cactaceae Cambial Zone Suggests Their Involvement in Wood Development
Source: Front Plant Sci. 2017 Mar 3;8:218. doi: 10.3389/fpls.2017.00218 (PMC5334636; doi:10.3389/fpls.2017.00218)

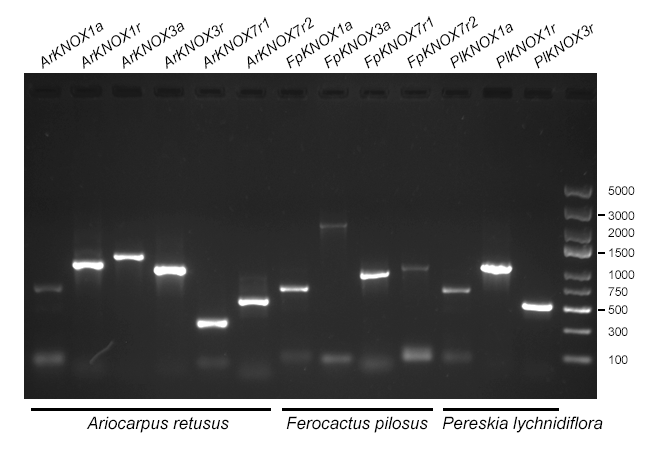

Supplement: FIGURE S1 — Class I and II KNOX transcript expression detected in cambial zone samples by end point RT-PCR. [file Image_1.TIF]

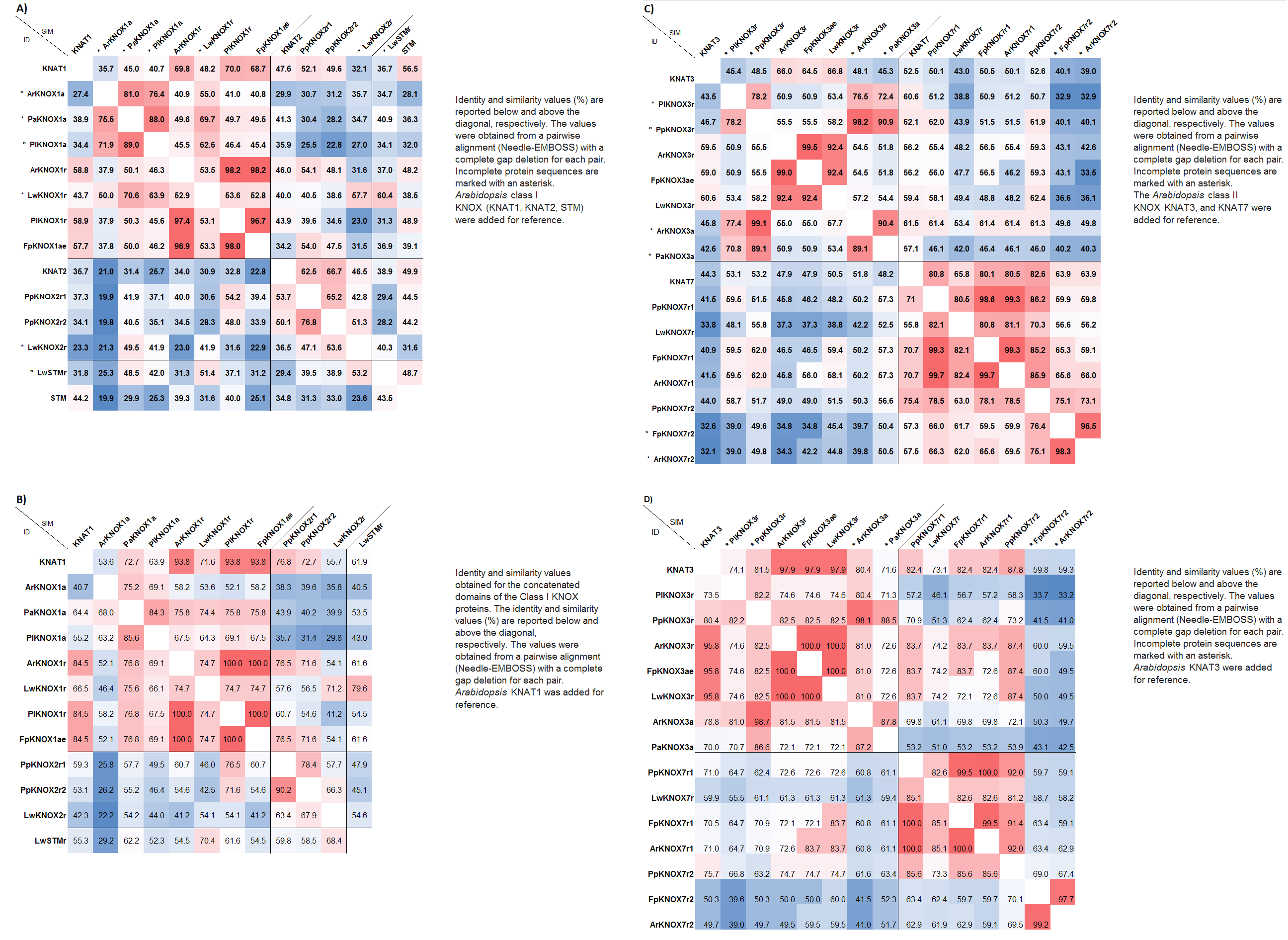

Supplement: FIGURE S2 — The identity and similarity matrix of the deduced KNOX proteins identified in this study. (A) Class I proteins. (B) Concatenated KNOX1, KNOX2, ELK, and HD domains of class I proteins. (C) Class II proteins. (D) Concatenated KNOX1, KNOX2, ELK, and HD domains of class II proteins. [file Image_2.TIF]
