# Supplementary material for: Expression of the KNOTTED HOMEOBOX Genes in the Cactaceae Cambial Zone Suggests Their Involvement in Wood Development
Source: Front Plant Sci. 2017 Mar 3;8:218. doi: 10.3389/fpls.2017.00218 (PMC5334636; doi:10.3389/fpls.2017.00218)
Supplement: TABLE S1 — PCR primers used in this study. [file Table_1.docx]

**Table S1. PCR primers used in this study.**

| Species | Gene | Primers | Amplicon Size |
| --- | --- | --- | --- |
| *Ariocarpus retusus* | *ArKNOX1r* | FWD: GGGAGGTTTCACCTATGTAACATC | 1026 |
|  |  | Rv: TCTTCAGATGGCTTCCAATGGC |  |
|  | *ArKNOX3r* | FWD: GCAACAGCTTCCTGAACCTTCAT | 954 |
|  |  | RV: TCTTGAGGGAAGTAGAACCAGAAG |  |
|  | *ArKNOX7r1* | FWD: AGAAGGCGAAACTGGTGGAG | 335 |
|  |  | RV: ATTCTTCTGGACCAAACAGTTGCC |  |
|  | *ArKNOX7r2* | FWD: ACTGCCCATGTTGCTTGTTTGA | 534 |
|  |  | Rv: TTTCCCAGCTCTTCTTTTCCTCAG |  |
| *Ferocactus pilosus* | *FpKNOX3ae* | FWD: CCCCCAACAAATGCTTCTTTCTTTC | 2738 |
|  |  | Rv: GGCTTCAACTCTAAGGGAGACTG |  |
|  | *FpKNOX7r1* | Fwd: ATGTTGGGTGTTTAAGGGTAGC | 940 |
|  |  | Rv: ATTCTTCTGGACCAAACAGTTGCC |  |
|  | *FpKNOX7r2* | Fwd: AGACTTACTTTCTCAGATCCCAACAAACCACC | 1032 |
|  |  | Rv: TATCACCCGGTAGTTTCCCAGCTCTTCTTTTC |  |
|  | *FpKNOX1ae** | Fwd:TTGAATCTGCGTAACCAAGTGCC | 205* |
|  |  | Rv:GTACTCCAAATAAAGGCCCTCAAC |  |
| *Pereskia lychnidiflora* | *PlKNOX1r* | Fwd: CTTCGCCTTCATCGTCGTCTTAT | 1065 |
|  |  | Rv: ACGTTCCAAATCGGTAAGGACC |  |
|  | *PlKNOX3r* | Fwd: TACAACAGCATGTCCGTGTCCATG | 530 |
|  |  | Rv: CTTGAGGGAAGTAGAACCAGAAGG |  |

* Primer pair used to confirm the expression of FpKNOX1 by the amplification of the 3'UTR region. The expected band was obtained but the result is not shown on the gel of Fig. S1.

The following PCR program was used:

|  |  |  | | 2 m |  | | 94 °C | |  |
| --- | --- | --- | --- | --- | --- | --- | --- | --- | --- |
|  |  |  | |  |  | |  | |  |
|  |  |  | | 35 s |  | | 94 °C | |  |
| **40 x** | | | | 35 s |  | | **56 °C** | |  |
|  |  |  | | 5 m |  | | 72 °C | |  |
|  |  |  | |  |  | |  | |  |
|  |  | | 10 m | | |  | | 72 °C | |
|  |  | | ∞ | | |  | | 4 °C | |

Expression of ArKNOX1a, ArKNOX3a, PlKNOX1a, and FpKNOX3ae transcripts was confirmed by RT-PCR with degenerate primers (for sequences see Material and Methods section). With degenerate primers, touchdown PCR program was used:

|  |  |  | 2 m |  | 94 °C |
| --- | --- | --- | --- | --- | --- |
|  |  |  |  |  |  |
|  |  |  | 30 s |  | 94 °C |
| **10 x** | | | 30 s |  | **62 °C** |
|  |  |  | 1 m |  | 72 °C |
|  |  |  |  |  |  |
|  |  |  | 30 s |  | 94 °C |
| **10 x** | | | 30 s |  | **60°C** |
|  |  |  | 1 m |  | 72 °C |
|  |  |  |  |  |  |
|  |  |  | 30 s |  | 94 °C |
| **15 x** | | | 30 s |  | **58 °C** |
|  |  |  | 1 m |  | 72 °C |
|  |  |  |  |  |  |
|  |  |  | 30 s |  | 94 °C |
| **10 x** | | | 30 s |  | **56 °C** |
|  |  |  | 1 m |  | 72 °C |
|  |  |  |  |  |  |
|  |  |  | 30 s |  | 94 °C |
| **10 x** | | | 30 s |  | **54 °C** |
|  |  |  | 1 m |  | 72 °C |
|  |  |  |  |  |  |
|  |  |  | 10 m |  | 72 °C |
|  |  |  | ∞ |  | 4 °C |
